# Defining Components of the ßcatenin Destruction Complex and Exploring Its Regulation and Mechanisms of Action during Development

**DOI:** 10.1371/journal.pone.0031284

**Published:** 2012-02-16

**Authors:** David M. Roberts, Mira I. Pronobis, Kelly M. Alexandre, Gregory C. Rogers, John S. Poulton, Daniel E. Schneider, Kuo-Chen Jung, Daniel J. McKay, Mark Peifer

**Affiliations:** 1 Department of Biology, University of North Carolina at Chapel Hill, Chapel Hill, North Carolina, United States of America; 2 Lineberger Comprehensive Cancer Center, University of North Carolina at Chapel Hill, Chapel Hill, North Carolina, United States of America; 3 Department of Biology, Franklin and Marshall College, Lancaster, Pennsylvania, United States of America; 4 Curriculum in Genetics and Molecular Biology, University of North Carolina at Chapel Hill, Chapel Hill, North Carolina, United States of America; 5 Department of Cellular and Molecular Medicine, University of Arizona, Tucson, Arizona, United States of America; National Cancer Center, Japan

## Abstract

**Background:**

A subset of signaling pathways play exceptionally important roles in embryonic and post-embryonic development, and mis-regulation of these pathways occurs in most human cancers. One such pathway is the Wnt pathway. The primary mechanism keeping Wnt signaling off in the absence of ligand is regulated proteasomal destruction of the canonical Wnt effector ßcatenin (or its fly homolog Armadillo). A substantial body of evidence indicates that SCF^βTrCP^ mediates βcat destruction, however, an essential role for Roc1 has not been demonstrated in this process, as would be predicted. In addition, other E3 ligases have also been proposed to destroy βcat, suggesting that βcat destruction may be regulated differently in different tissues.

**Methodology/Principal Findings:**

Here we used cultured *Drosophila* cells, human colon cancer cells, and *Drosophila* embryos and larvae to explore the machinery that targets Armadillo for destruction. Using RNAi in *Drosophila* S2 cells to examine which SCF components are essential for Armadillo destruction, we find that Roc1/Roc1a is essential for regulating Armadillo stability, and that in these cells the only F-box protein playing a detectable role is Slimb. Second, we find that while embryonic and larval *Drosophila* tissues use the same destruction complex proteins, the response of these tissues to destruction complex inactivation differs, with Armadillo levels more elevated in embryos. We provide evidence consistent with the possibility that this is due to differences in *armadillo* mRNA levels. Third, we find that there is no correlation between the ability of different *APC2* mutant proteins to negatively regulate Armadillo levels, and their recently described function in positively-regulating Wnt signaling. Finally, we demonstrate that APC proteins lacking the N-terminal Armadillo-repeat domain cannot restore Armadillo destruction but retain residual function in negatively-regulating Wnt signaling.

**Conclusions/Significance:**

We use these data to refine our model for how Wnt signaling is regulated during normal development.

## Introduction

Cell-cell signaling is critical for normal development and homeostasis. Key developmental signals can direct dramatic changes in cell fate, and thus in most signal transduction pathways, evolution has crafted high fidelity mechanisms to keep the pathway off in the absence of signaling. Regulated protein stability is often the control mechanism. Understanding in mechanistic detail how signaling effectors are stabilized or destroyed is thus critical to understanding signal transduction. Wnt signaling, which regulates cell fate decisions in virtually every tissue and organ in animals from fruit fly to human [1], provides a superb example. Wnt signals are transduced by stabilizing the effector ßcatenin (ßcat). Inappropriate activation of the pathway through failure to target ßcat for destruction underlies colon and other cancers [2].

In the current model of Wnt signaling [1], ßcat accumulates in cell-cell junctions in cells not receiving Wnt signal, where it has a distinct role in cadherin-based adhesion, but cytoplasmic ßcat levels are low. This is ensured by its short half-life. In the absence of signal, free ßcat is rapidly bound by a large multiprotein complex referred to as the destruction complex, in which the tumor suppressors APC and Axin bind ßcat. Axin also binds the kinases CKI and GSK3, facilitating sequential phosphorylation of ßcat's N-terminus. Phosphorylation creates a recognition site for E3-ubiquitin ligase using the F-box protein Slimb/ßTrCP, which targets ßcat for polyubiqitination and subsequent proteasomal destruction. When cells receive Wnt signals, receptor activation inactivates the destruction complex, by mechanisms whose details remain controversial. This stabilizes ßcat, which enters the nucleus and with TCF/LEF proteins activates Wnt target genes. Thus understanding regulated destruction of ßcat is key to understanding Wnt signaling.

SCF complexes are key E3 ubiquitin ligases [3], containing the substrate adaptor Skp1 (fly SkpA), the scaffold protein Cullin1, an F-box protein that binds substrate, and Roc1/Rbx1 (fly Roc1a), a RING-finger protein that recruits the E2 involved in ubiquitin transfer. A major advance in understanding ßcat regulation was the discovery that inactivating the *Drosophila* F-box protein Slimb (fly homolog of ßTrCP) prevents destruction of the fly ßcat homolog Armadillo (Arm) and activates Wnt signaling [4]. Published data also suggest roles for Skp1 and Cul1 in ßcat regulation, while Cul3, which uses BTB-domain proteins rather than F-box proteins as substrate adaptors, is not required [5].

However, two sets of data suggest that Arm degradation is more complex. First, although the Roc protein Roc1 is thought to be the RING finger component of all Cullin1-based SCF ligases, previous evidence suggested its fly homolog Roc1a is not essential for Arm degradation in wing imaginal discs, although it does mediate destruction of the Hedgehog effector Ci [6]. This suggests that additional E3 ligases may target Arm. One possibility is that a different RING-finger protein functions in Arm ubiquitination. This could be another Roc protein or a distinct RING-finger protein. Sina/Siah is a candidate; Siah can mediate p53-dependent βcat degradation, working with the F-box protein Ebi [7], [8]. Further, several other non-SCF-class E3 ligases have been suggested to regulate ßcat levels, including Jade-1/VHL [9], Cul4 [10], and Ozz/Cul5 [11]. The physiological roles of these alternate E3 ligases that target ßcat remains, in most cases, unclear, though in the case of Ozz, knockout mice suggest muscle specific roles. Thus we still must resolve which ubiquitin ligase(s) target Arm/ßcat for ubiquitination and whether all tissues use the same machinery for this task.

**Figure 1:**
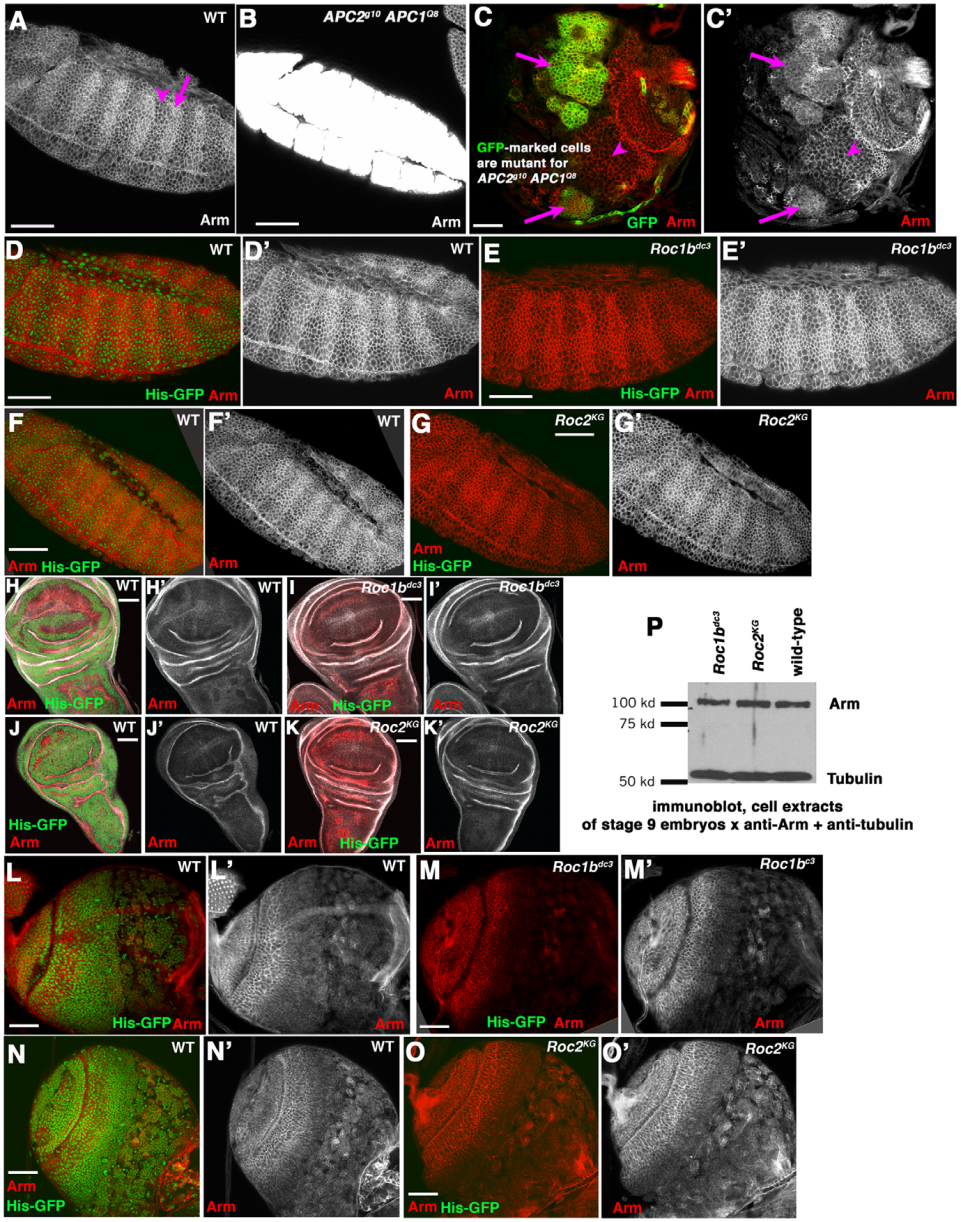
Neither Roc1b nor Roc2 is individually required for regulating Arm levels in embryos or larvae. Antigens and genotypes indicated. A–G. Embryos, anterior left. A. In wild-type stage 9–10 embryos segmentally repeated groups of cells receive Wingless signal, stabilizing Arm in the cytoplasm and nuclei (arrow). In other cells, Arm outside adherens junctions is destroyed (arrowhead). B. In *APC2 APC1* maternal/zygotic double mutant embryos, Arm levels are highly elevated, exceeding those in any cells in a wild-type embryo. C. When one induces clones of *APC2 APC1* double mutant cells in the developing larval brain (double mutant cells are marked with GFP using the MARCM technique), cytoplasmic Arm levels are modestly elevated (arrows) relative to wild-type cells (arrowhead). D–G. Stage 9 wild-type (D,F), *Roc1b^dc3^* maternal/zygotic (E), or *Roc2^KG^* maternal/zygotic embryos (G). For each mutant, wild-type embryos marked with Histone-GFP were stained in the same tube as mutants. Arm accumulation remains unchanged in both mutants. H–K. 3^rd^ instar wing imaginal discs from wild-type (H,J), *Roc1b^dc3^* zygotic mutants (I), or *Roc2^KG^* zygotic mutants (K). L–O. 3^rd^ instar larval brains from wild-type (L,N), *Roc1b^dc3^* zygotic mutants (M), or *Roc2^KG^* zygotic mutants (O). In both wing discs and brains no changes in Arm accumulation were apparent in either mutant. P. Immunoblot of cell extracts made from stage 9 embryos. Tubulin serves as a loading control. Scale bar = 50 µm.

A second puzzling issue regarding the identity of the machinery targeting Arm/ßcat for destruction in vivo comes from comparison of the roles of components of the destruction complex in different *Drosophila* tissues. Loss of Axin [12], [13], both APCs (Fig. 1A vs. B; [14], [15]), or GSK3 [16], [17] all lead to very high level Arm accumulation in *Drosophila* embryos. In contrast, loss of both APCs in the larval brain only subtly elevates Arm levels (Fig. 1C, arrows vs. arrowhead; [18]). This raised the possibility that different mechanisms may regulate Arm levels in different tissues and at different times.

A third issue concerns the mechanistic role of APC in the destruction complex. While a negative regulatory role has been clear for more than a decade, a recent study suggested that APC2 also has an unexpected positive role in Wnt signaling [19]. The mechanisms by which this occurs remain unclear, but certain *APC2* alleles retain the ability to positively regulate signaling while others do not.

We addressed these three issues, exploring which potential E3 ligase components regulate Arm levels in cultured cells and in vivo, particularly focusing on the role of Roc proteins, examining whether different regulatory mechanisms are at work in embryos and larvae, and exploring the functions in negative regulation of Wnt signaling by *APC2* alleles that do and do not retain the novel positive regulatory role.

## Results

### Assessing the roles of different Roc proteins in Arm regulation

A substantial amount of data support the idea that a Cullin1-based SCF complex with Slimb as the F-box protein regulates the targeted degradation of Arm/ßcat [4], [5], [20]. Flies have three Roc proteins—Roc1a associates with Cullins 1–4, Roc1b binds Cullin3, and Roc2 binds Cullin5 [21]. However, although Roc1a is a canonical component of the Cullin1-based SCF complex [21], *Roc1a* mutant clones in larval wing discs do not accumulate Arm above wild-type levels, but do accumulate a different SCF substrate, the Hedgehog effector Cubitus Interruptus [6]. Given these data, we set out to determine whether a different Roc protein in *Drosophila* acts in the SCF complex, or if the three Rocs function redundantly in this process.

We first tested these hypotheses by analyzing Arm accumulation in embryos and larval tissues lacking Roc1b or Roc2. We examined null alleles of *Roc1b* (*Roc1b^dc3^*, a coding sequence deletion that is homozygous viable but male sterile [22]) and of *Roc2* (*Roc2^KG^*, generated by P-element insertion, which is homozygous viable and fertile; [21]). We verified the presence of both mutations by PCR (data not shown). Given the essential role of Wnt signaling, the viability of *Roc1b* and *Roc2* mutants suggests that neither is an essential part of the E3 complex targeting Arm, or alternately suggests that the Roc proteins act redundantly.

To directly assess whether loss of either Roc1b or Roc2 affect Arm levels, we immunostained three tissues from *Roc1b^dc3^* or *Roc2^KG^* mutants (since both are viable, we could examine whole animals rather than clones of mutant cells). As an internal control, we stained wild-type animals marked with Histone-GFP together with each mutant, and imaged them on the same slides using the same confocal settings. In wild-type embryos, Arm is found at the plasma membrane of all epithelial cells, as part of the cadherin-catenin complex. In cells not receiving Wnt signal, there is little Arm inside cells, as it is targeted for destruction (Fig. 1A, arrowhead). Stripes of cells in each segment receive Wnt signals and accumulate Arm in the cytoplasm and nuclei (Fig. 1A, arrow). In contrast, embryos lacking the destruction complex proteins APC1 and APC2 accumulate Arm at very high levels, much higher than even wild-type cells receiving Wnt signal (Fig. 1B; [14], [15]). When compared to wild-type, neither *Roc1b^dc3^* mutant embryos (Fig. 1D′ versus E′) or *Roc2^KG^* embryos (Fig. 1F′ vs. G′) showed elevated Arm accumulation. We also did not see elevated Arm accumulation in imaginal discs mutant for either *Roc1b^dc3^* (Fig. 1H′ vs. I′) or *Roc2^KG^* (Fig. 1J′ vs. K′), or in larval brains mutant for either gene (Fig. 1L′ vs M′, 1N′ vs. O′). We also assessed Arm accumulation by immunoblot of protein from stage 9 embryos; *Roc1b^dc3^* and *Roc2^KG^* mutants have the same amount of Arm protein as wild-type (Fig. 1P). Together, these data suggest that neither Roc1b nor Roc2 is essential for regulating Arm degradation.

### An RNAi screen reveals SCF components regulating Arm stability in cultured *Drosophila* S2 cells

**Figure 2:**
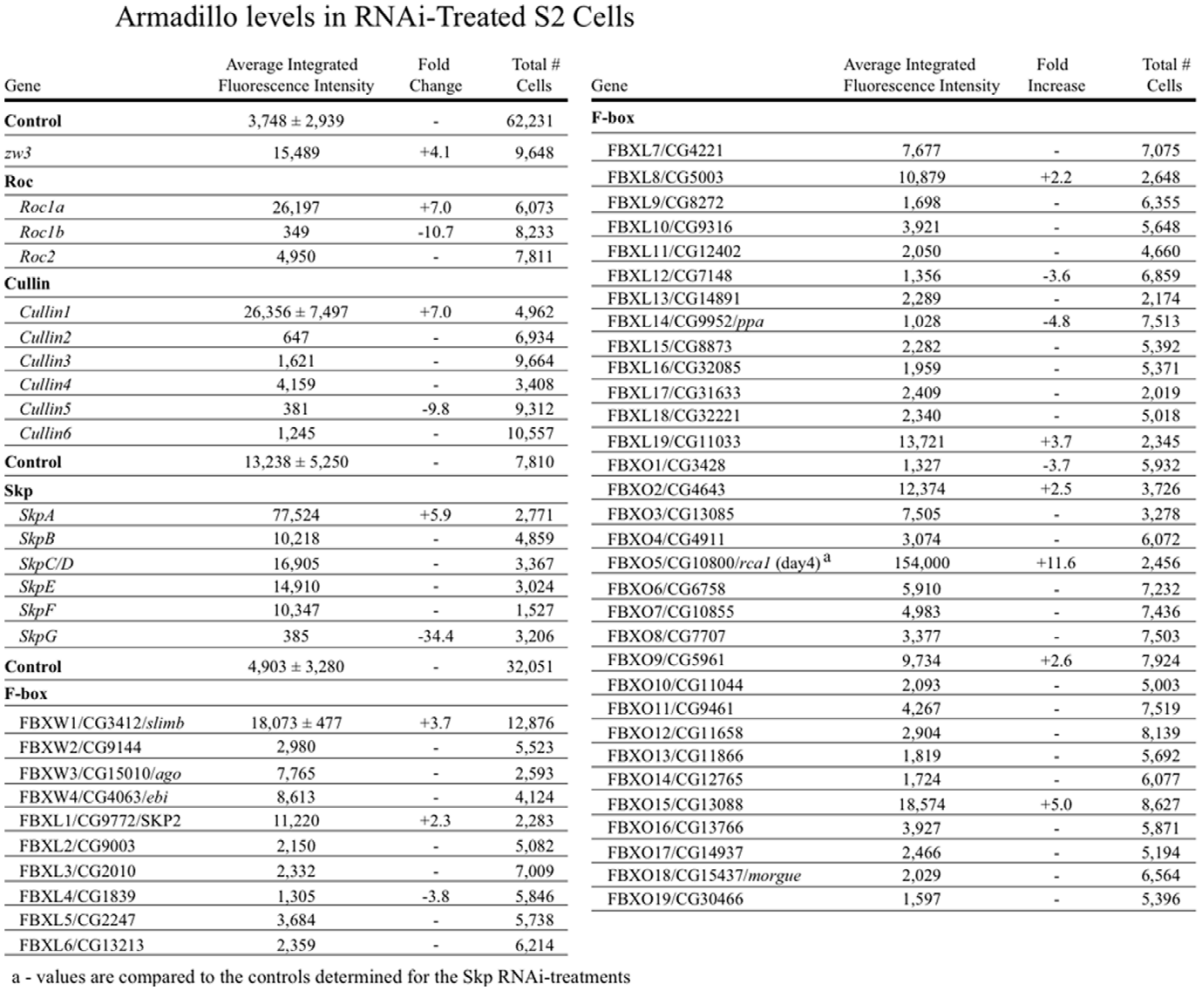
The results of our RNAi screen for SCF and E3 ligase components that alter Arm levels in *Drosophila* S2 cells.

Together with the earlier work on Roc1a in imaginal discs [6], these data suggest that none of the three Rocs are individually essential for Arm degradation, even though they are thought to be the key RING finger proteins in Cullin-based E3 ubiquitin ligases. We thus broadened our search for proteins regulating Arm stability, using an RNAi screen in *Drosophila* S2 cells. These cells are superb for this purpose: rather than having to design shRNAs and transfect them into cells, one simply adds ∼500 bp double-standed RNAs (dsRNAs) to the medium, and the cells take these up and process them into siRNAs. In parallel with a directed RNAi screen for SCF components that regulate centrosome number in cultured *Drosophila* S2 cells [23], we carried out a similar screen for proteins whose knockdown stabilized Arm. We examined the six fly Cullins, the seven fly Skp proteins, all three fly Rocs and a set of 42 F-box proteins. Cells were treated for 7 days with double-stranded RNA to each target protein in a multiwell format, and then fixed and stained for Arm, and also with Hoechst to label DNA to automate detection of individual cells. Plates then were scanned with an Array Scan V (Cellomics) automated microscope. Software was used to partition the field into cells, and images of 5000 cells per well were acquired and analyzed using vHCS View (Cellomics). This allowed us to quantitate Arm levels using average integrated fluorescence intensity (Fig. 2; several treatments reduced Arm levels—we did not pursue these further).

**Figure 3:**
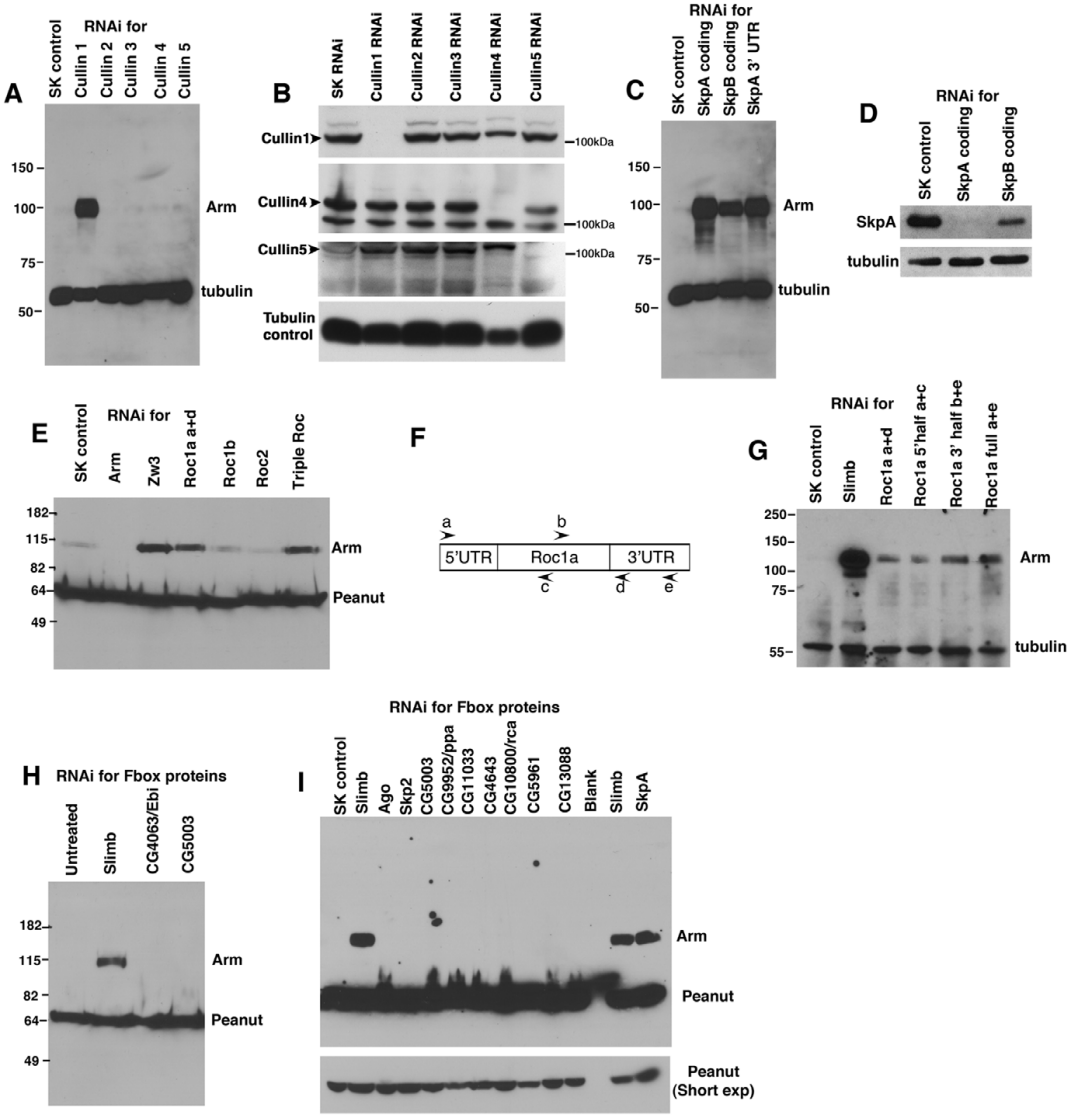
A canonical SCF complex including Roc1a regulates Arm levels in *Drosophila* S2 cells. Immunoblots of cell extracts from S2 cells treated with double-stranded RNA targeting the genes indicated. Tubulin and the septin Peanut serve as loading controls. The “SK” negative control is double-stranded RNA directed against the bacterial plasmid pBluescriptSK. A. Of the Cullins, only Cullin1 RNAi elevates Arm levels. B. Antibodies were available to confirm knockdown of Cullin1, Cullin4 and Cullin 5. All were significantly knocked down. These control samples were also used in the parallel screen for SCF proteins that regulate centrosome number, which was published in the Journal of Cell Biology [23]. C. RNAi directed against both SkpA and SkpB elevates Arm levels—to confirm the role of SkpA, we used RNAi directed against the non-conserved 3′ UTR. D. SkpB RNAi also reduces SkpA levels, presumably due to sequence similarity. E. Roc1a RNAi elevates Arm levels, as does triple RNAi against all three Rocs. SK RNAi serves as a negative control and RNAi against Zw3 (fly GSK3) as a positive control. F. Diagram of primers used to generate different dsRNAs against Roc1, some of which are non-overlapping, to test for off-target effects. G. RNAi against the 5′ or 3′ half of the Roc1a mRNA each lead to similar elevation of Arm levels as is caused by RNAi against the entire coding sequence. H,I. RNAi against each of the F-box proteins that scored positive in the primary screen, plus Ebi (which previously was reported to have a role in ßcat stability), Ago, and Ppa. Only Slimb RNAi elevated Arm levels.

Several genes scored positive for increased Arm levels. To follow-up these findings, these were examined more closely, by RNAi followed by immunoblotting for Arm. Cullin1, a core SCF complex component, was the only Cullin to score positive in the initial screen (Fig. 2). To followup, we repeated Arm immunoblots on cells treated with dsRNA to each of the five fly Cullins. Once again, Cullin1 was the only Cullin to score positive in the follow-up Western analysis, with knockdown elevating Arm levels (Fig. 3A). For three of the Cullins, Cullins1, 4 and 5, we were able to use available antibodies to verify knockdown in the same samples used to assess effects on Arm levels (Fig. 3B; these same controls were used to verify Cullin knockdown in our parallel screen for regulators of centrosome number [23]). This result is consistent with previous work in vivo suggesting a role for Cullin1 [5], but suggests that Cullin4, which has been reported to negatively regulate Arm/ßcat [10] in other contexts, is not a key regulator in *Drosophila* S2 cells. Among Skp proteins, only SkpA scored positive in the initial screen (Fig. 2). We did follow-up immunoblots for SkpA and SkpB; both scored positive for elevated Arm levels in this assay (Fig. 3C). However, due to sequence similarity between the two, SkpB knockdown also reduced SkpA levels (Fig. 3D). We suspect SkpA is the key player in vivo, as it is expressed at much higher levels than any of the other fly Skps [24]. Alternately, SkpA and SkpB may regulate Arm levels redundantly. Together, these data add further support to the model in which the primary E3 ligase targeting Arm for destruction is a canonical SCF complex using Cullin1 and SkpA.

The canonical SCF complex also uses the RING finger protein Roc1, but previous analysis in imaginal discs suggested the fly Roc1 ortholog (Roc1a) does not play a role in Arm regulation in that tissue [6]. However, in S2 cells our RNAi screen suggested Roc1a does play a role. RNAi of *Roc1a* substantially elevated Arm levels in the screen (Fig. 2). *Roc1a* RNAi also elevated Arm levels in the follow-up immunoblots (Fig. 3E). In contrast, neither RNAi of *Roc1b* nor *Roc2* alone elevated Arm levels in either assay (Fig. 2; Fig. 3E; *Roc1b* RNAi reduced Arm levels as assessed in the screen, perhaps due to subtle effects on cell cycle progression). Triple RNAi of all three Rocs also elevated Arm levels to approximately the same levels as *Roc1a* RNAi alone (Fig. 3E). Because of the discrepancy with earlier experiments on Roc1a in vivo, we carried out an additional experiment to ensure that the elevation of Arm levels in response to *Roc1a* RNAi was not due to an off-target effect of our original *Roc1a* dsRNA. We designed several different dsRNAs to *Roc1a*, including a pair of non-overlapping dsRNAs representing the 5′ and 3′ halves of the mRNA (Fig. 3F). Each of these led to elevated Arm levels relative to the SK RNAi control (Fig. 3G), consistent with our original result. Thus in S2 cells, Roc1a appears to be essential for Arm regulation, consistent with its known role in the SCF complex.

We tried several approaches to test whether Roc1a is essential for Arm degradation in the animal. One cannot make embryos maternally mutant for Roc1a as Roc1a is required for proliferation of germline stem cells [6]. We generated clones of *Roc1a* mutant cells in imaginal discs, but as was seen by Noureddine et al. (2002) [6], clones were infrequent and only comprised a few cells, and thus we could not effectively analyze Arm levels. We also tried using lines that were designed to allow in vivo *Roc1a* RNAi. We tested both a line from the Vienna RNAi collection [25], expressing it in imaginal discs, and a line from the Valium 20 collection [26], expressing it maternally using the matGAL4 driver. Neither effort produced either a change in Arm levels or any apparent phenotype (data not shown), suggesting that neither significantly depleted Roc1a—we have observed this with other RNAi lines from these collections. In the future additional RNAi lines may prove more effective, allowing our hypothesis to be tested in vivo.

The striking difference between the clear role we found for Roc1a in Arm destruction in S2 cells, and the failure to find such a role in imaginal discs [6] is consistent with two possibilities: 1) Roc1a may play a cell type specific role in Arm regulation, or 2) since loss of Roc1a is predicted to inactivate all SCF E3 ligases, it may be that when clones of *Roc1a* mutant cells are generated in imaginal discs [6], cells arrest due to effects on other target proteins before Roc1a levels drop severely enough to affect Arm regulation. Further work is needed to distinguish between these possibilities.

We next investigated which F-box proteins regulate Arm stability in S2 cells. Several F-box proteins scored at least marginally positive in our initial screen (Fig. 2)—we followed up each of these by repeating the RNAi and immunoblotting for Arm. The only F-box protein to score positive in both assays was the known Arm regulator Slimb (Fig. 3H,I; all lanes except SkpA are fly F-box proteins; most remain genetically uncharacterized and thus are only known by their CG numbers). It is also worth noting that we saw no effect on Arm levels in this cell type in either the screen or the follow-up immunoblots with RNAi against Ebi (Fig. 2; Fig. 3H), an F-box protein previously implicated in ßcat stability in other cell types [7], [8]. Of course Ebi and other F-box proteins may play roles in Arm/ßcat stability in a cell type specific manner, but they do not seem to play a critical role in Arm regulation in S2 cells.

### Is Armadillo regulation different in embryos and larvae?

**Figure 4:**
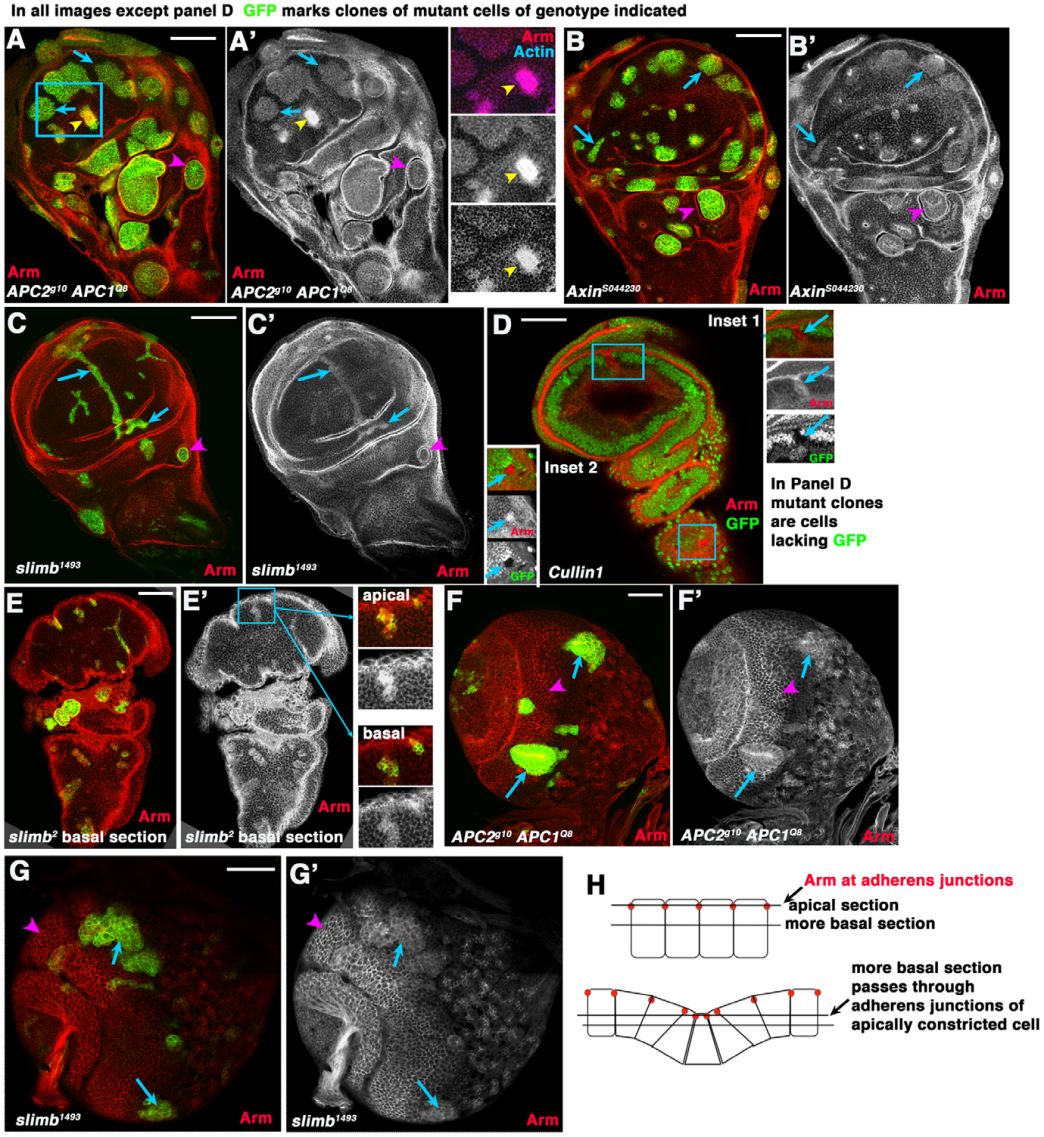
Arm accumulates to similar levels in wing imaginal disc cells mutant for different destruction complex or SCF proteins. A–E. 3^rd^ instar wing imaginal discs. F,G. 3^rd^ instar larval brains. In all cases except D clones of mutant cells of the indicated genotype were induced using the MARCM method [42] and homozygous mutant cells are marked by the presence of GFP. In D, homozygous mutant cells have lost GFP. A–C. Arrows, cells in the wing pouch mutant for both *APCs* (A), *Axin* (B) or *slimb* (C) all accumulate modestly elevated levels of Arm. Arrowheads, mutant cells in regions surrounding the wing pouch segregate and form cysts. A. Inset. Double mutant cells appearing to accumulate more elevated Arm levels are sectioned through the top of apically constricted cells, as demonstrated by their constricted apical ends and elevated actin accumulation in that plane of focus. D. We obtained very few and small clones mutant for Cullin1, which are marked by the lack of GFP—they accumulated elevated levels of Arm (Insets, mutant cells shown by arrows). E. *slimb* mutant cells, marked by GFP. Insets show an apical and more basal section through the same clone. Arm accumulation appears very high in apical section, but more basal section reveals more modest accumulation. Apical sections pass through the adherens junctions of mutant cells, which have apically constricted (diagrammed in H), creating the impression of more highly elevated Arm levels. F,G. Arrows, cells in the medulla mutant for both *APCs* (F) or *slimb* (G) accumulate modestly elevated levels of Arm. Arrowheads, wild-type cells showing normal levels of accumulation in this tissue. H. Diagrams illustrating changes in morphology in mutant clones and resultant effect on plane of focus in wild-type cells and mutant neighbors. Scale bars = 50 µm.

Another issue in the current literature about machinery regulating Arm levels during normal fly development concerns whether all tissues use the same machinery. This issue was raised by apparent differences between accumulation levels of Arm in embryos and larval tissues after inactivation of destruction complex or E3 ligase proteins. Arm accumulates to very high levels in fly embryos lacking both APC2 and APC1 (*APC2^g10^ APC1^Q8^* maternal zygotic mutants; (Fig. 1A vs. B; [14], [15]). In contrast, we previously found that clones of *APC2 APC1* double null mutant cells in the optic lobes of third instar larval brains only accumulate modest levels of Arm (Fig. 1C′, arrows vs. arrowheads, [18]). We first tested the hypothesis that this was a brain-specific difference, by examining Arm levels in clones of cells double mutant for null alleles of both *APC2* and *APC1* in third instar wing imaginal discs, relative to adjacent wild-type cells. As in the larval brain, apparent elevation of Arm levels was modest (Fig. 4A′, arrows; in this experiment and most of those below mutant cells are marked with GFP) relative to Arm elevation in double mutant embryos (Fig. 1A vs. B). As was previously observed [27], the activation of Wnt signaling in *APC2 APC1* double mutant cells also triggers a dramatic cell shape change. Cells apically constrict and invaginate to form cysts, particularly in regions surrounding the wing blade (Fig. 4A, arrowhead; [27]; activating Wnt signaling downstream of APC has similar effects [28]). These data suggest Arm levels are embryonic and imaginal cells are differentially sensitive to elimination of APC function.

Previous work demonstrated that wing imaginal disc cells mutant for *Axin* or *Slimb* accumulated elevated levels of Arm, helping demonstrate that these destruction complex or E3 ligase components are part of the machinery required to regulate Arm levels [4], [12]. The differential effect of loss of APC family proteins on relative Arm levels in embryos and imaginal discs led us to explore the hypothesis that there might be APC –dependent and APC-independent means of regulating Arm levels. To test this, we generated wing disc clones mutant for other destruction complex or E3 ligase proteins, including *Axin* and *slimb*, and directly compared Arm levels to those seen in *APC2 APC1* double mutant cells. As previously reported, immunostaining of wing discs revealed that clones mutant for *Axin* (Fig. 4B′, arrows) or *slimb* (Fig. 4C′, arrows) accumulate elevated levels of Arm. However, as we observed in *APC2 APC1* double mutant cells, (Fig. 4A′, arrows), the elevation of Arm levels in *Axin* or *slimb* mutant cells was not as extreme as that previously seen in embryos lacking destruction complex proteins. Loss of Slimb in clones of cells in the larval brain optic lobe also only resulted in modest elevation of Arm levels (Fig. 4G′), qualitatively similar to what we observed in cells double mutant for both APCs (Fig. 4F′; [18]). We also saw elevated Arm levels in the few wing imaginal disc clones mutant for *Cullin1* we obtained (Fig. 4D, insets—note that here mutant cells are those lacking GFP). Cullin1 clones were very small and rare, probably due to effects on other SCF targets important for cell viability or cell cycle progression; similar clone size and rarity were previously seen in clones mutant for Roc1a [6]. We also noted in passing that cells mutant for *Axin* (Fig. 4B, arrowhead) or *slimb* (Fig. 4C, arrowhead) also invaginated, forming cysts like those seen with *APC2 APC1* double mutants [27]. Thus, disruption of different components of the destruction complex or the E3 ligase in larval tissues led to similar modest elevation of Arm levels, reducing the likelihood of an APC-independent mechanism of Arm regulation.

In previous work [4], [12] and in our own data, a subset of clones mutant for *slimb*, *Axin*, or double mutant for *APC2 APC1* did appear to accumulate highly elevated levels of Arm (e.g., Fig. 4A, arrowhead). We thus explored the reason for this apparent discrepancy. As noted above, in addition to affecting Arm levels and activating Wnt target genes, activating Wnt signaling in clones of cells in imaginal discs has drastic consequences for cell morphology—cells with activated Wnt signaling apically constrict, distorting the epithelial sheet [27], [28]. This can be clearly seen in some clonal patches, where co-staining with actin reveals groups of mutant cells with strongly constricted apical ends (Fig. 4A, yellow arrowhead in boxed region, yellow arrowhead in inset). Both actin and Arm are strongly enriched in cell-cell adherens junctions [29], which are in the apical-most region of the lateral cell membrane. We thus hypothesized that the apparent high level of accumulation in mutant clones such as these might be due to differences in the plane of focus between wild-type cells and adjacent mutant neighbors, due to changes in the folding of the epithelial sheet. Images taken at the apical-most end of even a wild-type cell will show a higher level of Arm than a more basal section, because the apical-most section will pass through the adherens junction (Fig. 4H, top). Consistent with the hypothesis that differences in apparent Arm accumulation could be caused by differences in cell morphology, Arm staining was relatively brighter in *APC2 APC1* double mutant clones which have apically constricted (e.g., Fig. 4A, blue arrows are non-apically constricted cells versus yellow arrowhead showing apically constricted cells, as revealed by the bright actin staining of the constricted cells). To further test this hypothesis, we examined different sections through clones mutant for *slimb*. In fact, sections through the same clone revealed apparently very high levels of Arm in mutant clones in very apical sections (Fig. 4E′, top inset), while a more basal section of the same clone has more modest elevation of Arm (Fig. 4E′, bottom inset)— likely because more apical sections pass through adherens junctions of apically constricted mutant cells and more basal regions of neighboring wild-type cells (Fig. 4H, bottom). Thus together, our data support the idea that the same machinery regulates Arm levels in embryonic and larval tissues. However, the consequences of removing this machinery on Arm levels differ between the tissues.

We next addressed the question of why we observed such a striking difference in Arm accumulation after destruction complex inactivation when comparing embryos and larval tissues. We hypothesized that in embryos the known transcriptional up-regulation of *arm* after the midblastula transition [30] might program the translation of more Arm protein, but that this newly synthesized protein might be rapidly turned over by the destruction complex. In this hypothesis, since cells in stage 9 embryos would have higher levels of *arm* mRNA than cells in larval tissues, they would respond to inactivating the destruction complex by accumulating Arm protein more rapidly.

**Figure 5:**
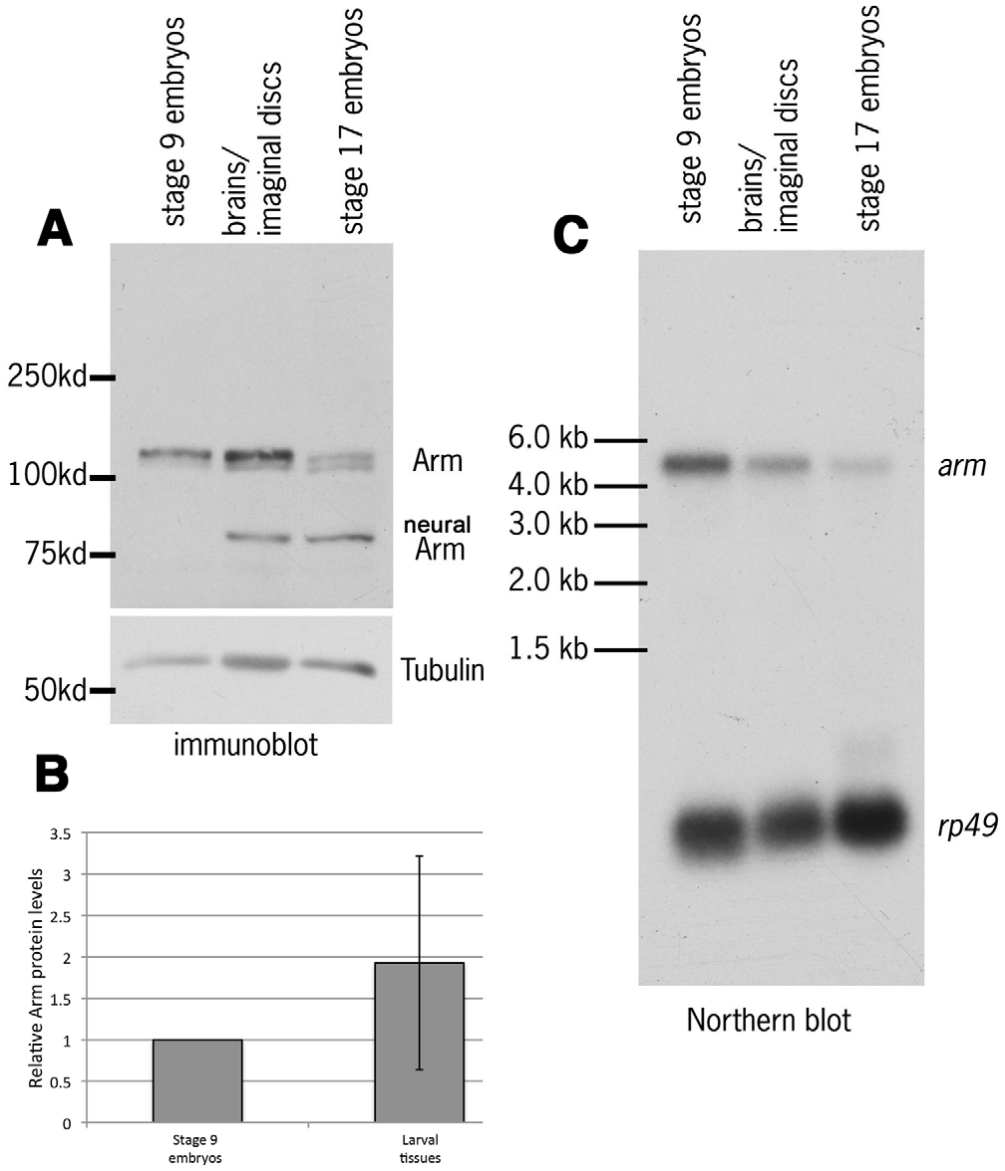
While Arm protein levels are similar in larval imaginal tissues and stage 9 embryos, *arm* mRNA is more abundant in stage 9 embryos. A. Immunoblot of cell extracts from stage 9 or stage 17 embryos, or from 3^rd^ instar brains and attached imaginal discs. Note that Arm is differentially spliced in neurons to produce a shorter form, neural Arm (nArm; [46]), which is present in stage 17 embryos and in the brain. B. Arm protein in larval imaginal tissues is at levels similar to those in stage 9 embryos, when normalized to tubulin as a loading control (the two tissues were not significantly different when compared by a one sample t test; p = 0.34). C. Northern blot of total RNA from stage 9 and stage 17 embryos and from 3^rd^ instar brains and attached imaginal discs. Densitometry revealed that *arm* mRNA is present at about 2 fold the level in stage 9 embryos than it is in larval imaginal tissues, when normalized to the ribosomal protein mRNA *rp49*.

This hypothesis predicts that the ratio of *arm* mRNA to protein would be higher in stage 9 embryos than in larval tissues. To test this hypothesis, we first compared Arm protein levels (Fig. 5A) of stage 9 embryos (when Wnt signaling is maximal), wing discs and brains of third instar larvae, and, as a control, stage 17 embryos (after most Wnt signaling in embryos is done and when we expected Arm protein levels to be low; [31]). Arm protein accumulation increases in stage 9 embryos as segment identities are defined [31]. We found that the amount of Arm was not significantly different in larval tissues than in stage 9 embryos, when normalized to tubulin (Fig. 5A; quantified in Fig. 5B). Next, we looked at *arm* mRNA levels, comparing mRNA levels from wild-type animals from all three stages by Northern blot, using the ribosomal protein gene *rp49* as a loading control (Fig. 5C). *arm* mRNA levels in stage 9 embryos were roughly two times higher than in 3^rd^ instar larval brains and imaginal discs, when normalized to the *rp49* (Fig. 5C; *arm* mRNA levels were even lower in stage 17 embryos, as expected [30]). To confirm this and deal with the issue that our Northern analysis combined both imaginal discs and brains, we used RNAseq data from hand-dissected imaginal discs (Table 1). Using the same normalization to *rp49*, we found that *arm* transcripts were 2.6 fold more abundant in stage 9 embryos than in 3^rd^ instar wing imaginal discs. Together, these data suggest that there is more *arm* mRNA in embryos than in larval tissues, despite similar levels of protein. Thus if levels of translation are equivalent, the destruction complex would have to destroy more newly synthesized Arm in stage 9 embryos than in larval tissues. This model further predicts that if the destruction complex were inactivated, Arm levels would increase more dramatically in embryos than in imaginal tissues, which is in fact what we observed.

**Table 1:** RNAseq transcript numbers for *arm* normalized to *rp49*.

Gene	6–8 hour embryo	16–18 hour embryo	3^rd^ instar wing imaginal disc
*arm*	4849.99	1863.93	2171.5
*rp49 = rpl32*	17976	14519.4	25805.6
*arm/rp49*	0.270	0.128	0.0841

This hypothesis is also consistent with previous work on *APC2* alleles of different strengths. Both null and hypomorphic alleles cause significant effects on cell fate in the embryo [32], though they differ in the strength of these effects. In contrast, null and hypomorphic *APC2* alleles have very different effects in the imaginal discs. In clones of cells double mutant for null alleles of *APC2* and *APC1*, Wnt target genes are activated, and cells apically constrict and invaginate, and those that do not apoptose ultimately exhibit fate changes in the adult wing, taking on wing margin fates [27]. In contrast, in clones of cells double mutant for hypomorphic *APC2* alleles and a null allele of *APC1*, all these phenotypes are reduced or eliminated [27]. These data suggest that cells in larval wing imaginal discs require less APC2 function to regulate the Wnt pathway than do cells in stage 9 embryos, consistent with the different levels of destruction complex activity predicted to be required from the higher levels of *arm* mRNA in embryos than in larval tissues.

### *APC2^33^* is hypomorphic and retains residual function in embryos, imaginal discs and the larval brain

This difference in phenotype between null and hypomorphic alleles in wing imaginal discs also allowed us to further characterize two interesting alleles of APC2. In 2008, Takacs et al. [19] described a series of experiments suggesting that APC2 in the developing *Drosophila* eye had paradoxical effects—reducing levels of APC2 suppressed the effects of inappropriate Wnt activation caused by loss of APC1, suggesting APC2 might have positive as well as negative roles in Wnt signaling [19].

**Figure 6:**
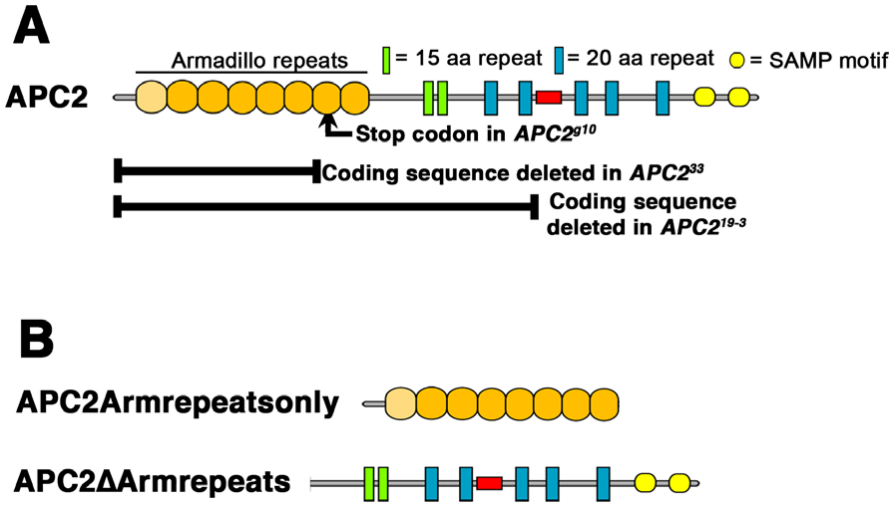
Mutations in *APC2^g10^*, *APC2^33^* and *APC219-3*, and structure of APC2ΔArmrepeats and APC2Armrepeatsonly.

During their analysis, they found that different *APC2* alleles they tested differed in whether they suppressed loss of APC1. Surprisingly, the allele we standardly use as a null allele, *APC2^g10^*, did not suppress effects of APC1 loss, although deletion of the genomic region including *APC2* did so [19]. This was surprising, as *APC2^g10^* has a stop codon about one-third of the way through the coding sequence (in the seventh Arm repeat; Fig. 6A), and we could not detect a truncated protein with an N-terminal antibody [32], although we could detect a truncated protein in an allele with a slightly later stop codon [32]. In contrast, effects of loss of APC1 were suppressed by two new alleles of *APC2* that were generated by mobilizing transposable elements in the 5′ flanking region or 5′ UTR [19]. Both deleted part *APC2's* coding sequence— *APC2^19–33^*deletes the translation start and most of the coding sequence, including all the Arm repeats, the 15 amino acid repeats, and the first two 20 amino acid repeats (Fig. 6A), while *APC2^33^* deletes the transcription and translation starts and coding sequence extending into the 5th Arm repeat (Fig. 6A). Based on differences in Arm accumulation in imaginal discs between cells double mutant for *APC2^33^* and a null allele of *APC1* versus cells double mutant for definitive null alleles of both *APC2* and *APC1*, they suggested that *APC2^33^* might encode an N-terminally truncated APC2 protein lacking most of the Arm repeats, but retaining the 15 and 20 amino acid repeats that bind Arm/ßcat and the SAMP repeats that bind Axin, and also retaining some function in negatively regulating Wnt signaling (it is worth noting that they could not detect this protein by immunoblotting [19], so its levels must be very low). Consistent with this, recent work revealed that remnant mobile elements like those remaining at the site of deletion in both alleles [19] can contain promoters driving expression of adjacent genes [33]. Since N-terminally truncated fragments of human APC can rescue ßcat degradation in human colon cancer cells [34], it is not inconceivable that *APC2^33^* or even *APC2^19–33^* might encode very low levels of an N-terminally truncated APC2 protein that nonetheless retained some function in Wnt regulation. Takacs et al. thus suggested that our allele *APC2^g10^* produced very low levels of a C-terminally truncated APC2 that retained some residual activity in negatively regulated signaling, and also retained the postulated positive effect of APC2 on Wnt signaling, while the putative N-terminally truncated APC2 protein produced by *APC2^33^* lacked this positive effect of APC2 on Wnt signaling.

**Figure 7:**
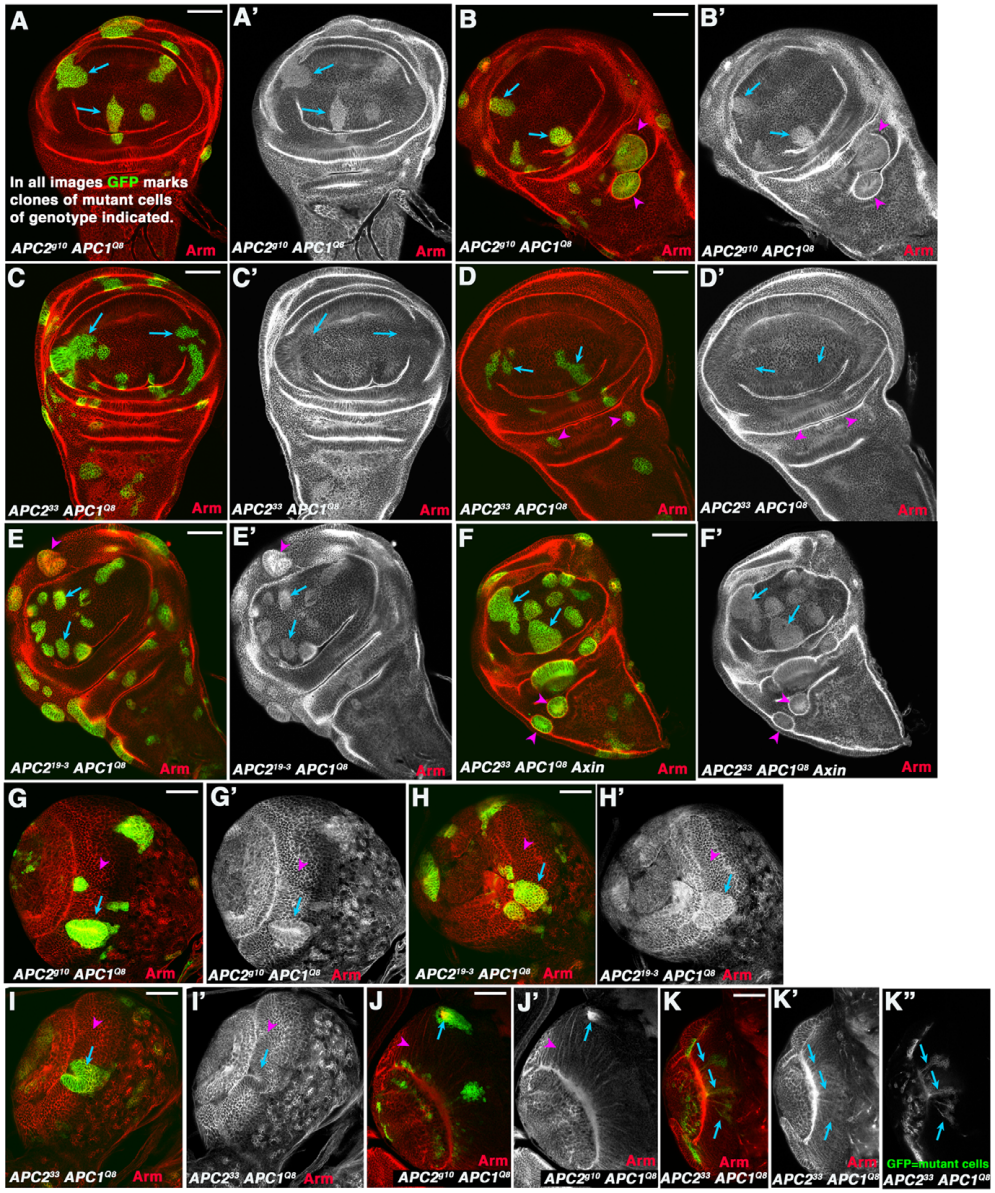
*APC2^33^* has a hypomorphic phenotype. A–F. 3^rd^ instar wing imaginal discs. G–K. 3^rd^ instar larval brains. Clones of mutant cells of the indicated genotype were induced using the MARCM method [42] and homozygous mutant cells are marked by the presence of GFP. A–B. Cells in the wing pouch that are *APC2^g10^ APC1^Q8^* double mutant accumulate modestly elevated levels of Arm (arrows), while mutant cells in regions surrounding the wing pouch segregate and form cysts (arrowheads). C,D. In contrast, cells in the wing pouch that are *APC2^33^ APC1^Q8^* double mutant do not accumulate elevated levels of Arm (arrows), and mutant cells in regions surrounding the wing pouch do not always segregate to form cysts (arrowheads). E,F. Clones of cells that are *APC2^19-3^ APC1^Q8^* double mutant (E) or *APC2^33^ APC1^Q8^ Axin* triple mutant (F) behave like *APC2^g10^ APC1^Q8^* double mutant cells. G. Neurepithelial cells in anterior medullar region of the larval brain that are *APC2^g10^ APC1^Q8^* double mutant accumulate modestly elevated levels of Arm (arrow) and segregate from neighbors, in contrast to neighboring wild-type cells (arrowhead; [18]). H. Neurepithelial cells in anterior medullar region of the larval brain that are *APC2^19-3^ APC1^Q8^* double mutant behave similarly to *APC2^g10^ APC1^Q8^*. I. Neurepithelial cells in anterior medullar region of the larval brain that are *APC2^33^ APC1^Q8^* double mutant sometimes segregate but do not always accumulate elevated Arm levels (arrow vs. arrowhead). J. Medullar neurons that are *APC2^g10^ APC1^Q8^* double mutant invariably send out axons into the center of the clone, forming axonal knots (arrow; [18]) instead of the normal finely fasciculated projections (arrowhead) to the medullar neuropil [18]. K. Some medullar neurons that are *APC2^33^ APC1^Q8^* double mutant do not form axonal knots but instead send normal projections to the medullar neuropil (arrows). Scale bars = 50 µm.

We used imaginal discs to directly compare the effects on Wnt regulation of three different *APC2* alleles, which had distinct effects in the assays of Takacs et al. [19]. To do so, we assessed Arm levels and cell behavior in clones of cells double mutant for each of these different alleles *APC2* and also mutant for a definitive null allele of *APC1*, *APC1^Q8^* (with a stop codon in Arm repeat 4; [35]). Cells double mutant for *APC2^19-3^* and *APC1^Q8^* (Fig. 7E) resembled cells double mutant for our standard null allele *APC2^g10^* and *APC1^Q8^* (Fig. 7A,B). In both cases mutant cells in the wing pouch accumulated elevated levels of Arm (Fig. 7A,B,E, arrows), and cells around the margin of the wing pouch also apically constricted and invaginated (Fig. 7A,B,E arrowheads). In contrast, as reported by Takacs et al., cells double mutant for *APC2^33^* and *APC1^Q8^* did not accumulate detectably elevated levels of Arm (Fig. 7C,D, arrows), nor did they invaginate from the imaginal disc epithelium (Fig. 7C,D arrowheads). In contrast, cells triple mutant for *APC2^33^*, *APC1^Q8^*, and *Axin* did accumulate Arm (Fig. 7F, arrows), showing that there was not a suppressor of this phenotype on the chromosome. In its properties *APC2^33^* resembles other previously characterized hypomorphic *APC2* alleles [27]. These data are thus consistent with the possibility that *APC2^33^* produces an N-terminally truncated protein retaining some function in negatively regulating Wnt signaling, while suggesting that *APC2^19-3^* is a functional null allele.

We saw similar differences between *APC2^33^* and the other two *APC2* alleles when we examined clones of *APC2 APC1* double mutant cells in the larval brain. As we previously observed [18], clones of cells in the medullar region of the brain that are double mutant for our standard null allele *APC2^g10^* and *APC1^Q8^* accumulate modestly elevated levels of Arm, and segregate from their neighbors (Fig. 7G, arrow versus arrowhead); when clones are generated in medullar neurons, their axons do not extend to the medullar neuropil and instead form knots in the center of the clones. Cells double mutant for *APC2^19-3^* and *APC1^Q8^* behaved similarly, accumulating elevated Arm levels and segregating from their neighbors (Fig. 7H, arrow vs. arrowhead). In contrast, *APC2^33^ APC1^Q8^* double mutant cells exhibited a weaker phenotype—while double mutant medullar neurepithelial cells sometimes segregated from their neighbors (Fig. 7I, arrow), Arm accumulation was less obvious. Further, while *APC2^g10^ APC1^Q8^* double mutant neurons send out axons into a knot in the center of the clone (Fig. 7J, arrow; [18]), *APC2^33^ APC1^Q8^* double mutant neurons did not form axon knots, but instead sent axons to the medullar neuropil (Fig. 7K, arrows) as do wild-type neurons [18]. In these ways *APC2^33^* behaved similarly to other hypomorphic *APC2* alleles [18]. Finally, we examined embryos maternally and zygotically *APC2^33^*mutant, using cuticle preparations to assess the strength of defects in Wnt signaling by the numerical scale of McCartney et al [31], where 0 is a wild-type embryo and 6 indicates the most severe defects. *APC2^33^* maternal/zygotic mutants had an average cuticle score of 3.2 (n = 251). This is less severe than *APC2^g10^*, and is in the range of other hypomorphic mutants [32]. Together these data further support the hypothesis of Takacs et al. that *APC2^33^* is hypomorphic and not null for negative regulation of Wnt signaling. They also reinforce the idea there is not a one-to-one correspondence between the negative regulatory effects of a given *APC2* allele on Wnt signaling and its ability to suppress loss of APC1—both *APC2^g10^* and *APC2^19-3^* have stronger effects on Wnt regulation than *APC2^33^*, yet only *APC2^19-3^* and *APC2^33^* suppress the loss of APC1.

### An APC2 protein lacking the Arm repeats retains residual activity in Wnt regulation

**Figure 8:**
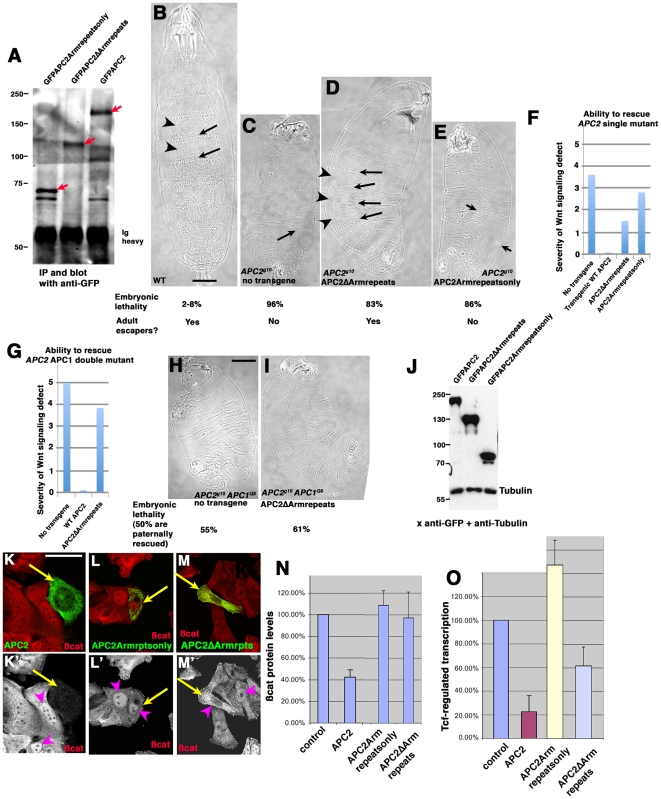
APC2 lacking its Arm repeats cannot downregulate ßcat levels but retains some ability to blunt Wnt signaling. A. The mutant proteins accumulate at near normal levels. Protein from embryo extracts expressing wild-type GFPAPC2, GFPAPC2ΔArmrepeats, or GFPAPC2Armrepeatsonly was immunoprecipitated with anti-GFP antibodies, separated by SDS-PAGE, and immunoblotted with anti-GFP antibodies. The expected transgenic proteins are indicated by red arrows and antibody heavy chain is also labeled. B–E. Representative cuticles from wild-type, and embryos maternally and zygotically null mutant for *APC2*, either alone or expressing the indicated transgene. Embryonic lethality and presence of adult escapers indicated below. B. Wild-type cuticle, showing alternating anterior cells secreting denticles (arrows) and posterior cells secreting naked cuticle (arrowheads). C. In *APC2^g10^* maternal/zygotic mutants, almost all cells are converted to posterior fates and only a few cells secrete denticles (arrow). D. APC2ΔArmrepeats restores alternately denticle belts (arrows) and naked cuticle (arrowheads), though denticle belts are often incomplete. E. In *APC2^g10^* maternal/zygotic mutants expressingAPC2Armrepeatsonly, most cells remain transformed to posterior fates and only a few cells secrete denticles (arrow). F. Quantification of rescue of Wnt signaling defects of embryos maternally and zygotically null mutant for *APC2* by a GFP-tagged wild-type *APC2* transgene (scoring scheme and wild-type rescue data from [36]), or by transgenes encodingAPC2ΔArmrepeats, or APC2Armrepeatsonly. G. Quantification of rescue of Wnt signaling defects of embryos maternally and zygotically double null mutant for *APC2* and *APC1* by either a GFP-tagged wild-type *APC2* transgene (scoring scheme and wild-type rescue data from [36]) or by APC2ΔArmrepeats. H,I. Representative cuticles and embryonic lethality. Since the lethality of embryos expressing APC2ΔArmrepeats is higher than that of embryos with no transgene, this suggests additional embryos that are paternally-rescued may be dying, perhaps due to some dominant-negative activity of this protein on the paternally contributed APC2. Thus even the subtle degree of apparent rescue may simply reflect averaging in the less severe phenotype of these additional paternally rescued embryos. J. All transgenes are expressed and accumulate stably in SW480 cells. Immunoblot of cell extracts of human SW480 cells transfected with the indicated constructs. All of the APC2 constructs are N-terminally GFP tagged and detected with anti-GFP antibody. Tubulin serves as a loading control. K–M. SW480 cells transfected with the indicated constructs. GFP and ßcat. Arrows indicate transfected cells. K. SW480 cells, which are mutant for human *APC*, accumulate high levels of ßcat in their cytoplasm and nuclei (arrowhead). Transfection with fly APC2 rescues ßcat destruction (arrow). L. APC2Armrepeatsonly (arrow) does not rescue ßcat destruction or its nuclear localization. M. APC2ΔArmrepeats (arrow) does not rescue ßcat destruction but can retain some ßcat in the cytoplasm, lowering levels in nuclei (compare arrowheads). N. Only wild-type APC2 reduces ßcat levels, as quantified by Cellomics. O. Wild-type APC2 strongly reduces expression of the Wnt-regulated reporter gene, TOPFLASH, APC2ΔArmrepeats reduces TOPFLASH somewhat, and APC2Armrepeatsonly does not reduce TOPFLASH. Scale bars = 50 µm).

These data and those of Takacs et al. suggested the hypothesis that APC2 proteins lacking the Arm repeats might retain some function in Wnt regulation. However, this was based on the hypothetical N-terminally protein encoded by *APC2^33^* , which Takacs et al. could not detect by immunoblotting [19]. To directly explore the function of such an N-terminally truncated APC2 protein, we generated a GFP-tagged mutant of APC2 largely matching the protein that might be produced by *APC2^33^*. We expressed it using its own ATG codon and from the endogenous *APC2* promoter and verified accumulation levels were near normal, relative to wild-type GFP-APC (Fig. 8A). This mutant, APC2ΔArmRepeats, lacks the Arm repeats but retains the 15 and 20 amino acid repeats and SAMP repeats (Fig. 6B). In parallel, we generated a mutant encoding only the Arm repeats of APC2 (APC2Armrepeatsonly; Fig. 6B; 8A), which should largely mimic hypothetical predicted protein made by *APC2^g10^*.

We then tested whether these two proteins could negatively regulate Wnt signaling, using transgenic flies in which the mutant proteins were expressed at normal levels under control of the endogenous promoter [36]. We explored their ability to rescue Wnt signaling in the embryonic epidermis, using the cuticle as a measure. Anterior cells in wild-type embryos secrete hair-like denticles (Fig. 8B, arrows), while posterior cells secrete naked cuticle (Fig. 8B, arrowheads). We first tested APC2ΔArmRepeats in embryos maternally and zygotically null for *APC2*. These embryos have strong Wnt pathway activation, but retain a small amount of Wnt regulation due to the low levels of APC1 remaining [14], [15]. As a result almost all cells are converted to posterior fates and only a few denticles remain (Fig. 8C). When we expressed APC2ΔArmRepeats in the *APC2^g10^* maternal/zygotic mutant, it significantly rescued Wnt signaling in the embryonic epidermis (Fig. 8D, quantified in 7F), largely but not completely restoring anterior cell fates and thus denticle belts to the cuticle. In contrast, APC2Armrepeatsonly had only a modest rescuing effect (Fig. 8E,F). We next tested APC2ΔArmRepeats in maternal and zygotic *APC2 APC1* double mutant embryos. In these embryos all cell fates are converted to naked cuticle (Fig. 8H; [14], [15]). This is a more stringent test of the activity of the mutant protein [32], [36]. In this background, APC2ΔArmRepeats provided only very weak rescuing activity (Fig. 8I; quantified in 8G), contrasting with its stronger rescuing ability in the single *APC2* mutant. Based on comparison with other mutants we have analyzed [36], this suggests that APC2ΔArmRepeats cannot rescue Arm degradation, but may be able to blunt Wnt signaling by sequestering Arm.

To test this directly, we assessed both mutants in cultured human SW480 colon cancer cells, which carry a truncated version of human APC, and thus accumulate very high levels of ßcat in the cytoplasm and nucleus [34]. We previously found that *Drosophila* APC2 effectively rescues Wnt regulation in these cells, reducing both ßcat levels and Wnt-regulated transcription [36]. We thus transfected SW480 cells with GFP-tagged *Drosophila* APC2, APC2ΔArmRepeats, or APC2Armrepeatsonly. We confirmed expression of stable proteins both by immunoblotting cell extracts with anti-GFP antibody (Fig. 8J; tubulin was the loading control), and by GFP-fluorescence in transfected cells (Fig. 8K–M). Wild-type fly APC2 reduces ßcat levels in these cells [36], as assessed by immunofluorescence (Fig. 8K) or by automated quantitation of hundreds of cells (Fig. 8N). In contrast, neither APC2ΔArmRepeats nor APC2Armrepeatsonly down-regulated ßcat levels by either assay (Fig. 8L–N, transfected cells are marked with GFP). However, APC2ΔArmRepeats (but not APC2Armrepeatsonly) could reduce expression of the Wnt-responsive reporter TOPFLASH (Fig. 8O). When we compare these results to those we saw with a series of other mutants in APC2 we tested [36], the phenotypes of APC2ΔArmRepeats fit best with mutant proteins that cannot not rescue Arm/ßcat destruction, but, because they retain ßcat binding sites, can sequester ßcat in the cytoplasm and thus reduce downstream Wnt signaling. Our immunofluorescence images of APC2ΔArmRepeats are consistent with this hypothesis—expression of this mutant somewhat reduced relative ßcat levels in the nucleus (Fig. 8M, compare arrowheads). Together, these data suggest that an APC2 protein lacking the Arm repeats can blunt Wnt signaling somewhat, and are consistent with the idea that the hypothetical truncated *APC2^33^* protein might act similarly, helping explain its hypomorphic phenotype in imaginal discs.

## Discussion

Arm/ßcat is the key effector of canonical Wnt signaling. Properly regulating its stability is thus essential for normal development and adult homeostasis, and mis-regulation of ßcat stability is implicated in colon and other cancers. Here we address several questions raised by the current literature concerning the normal regulation of Arm/ßcat stability. We assessed components of the E3 ubiquitin ligase(s) targeting Arm for destruction in *Drosophila* S2 cells and in fly tissues, explored whether Arm stability is differentially regulated in embryos and larval tissues, and investigated the function of APC2 proteins lacking their Arm repeats in regulating Wnt signaling.

### Roc1a is required to regulate Arm stability

Previous work strongly supported the idea that a canonical SCF complex using Slimb/ßTrCP as a substrate recognition factor is the primary means of regulating Arm stability [4], [5]. However, there was one major discrepancy in the literature that disagreed with this model. Roc proteins are the RING-finger proteins in Cullin-based E3 ligases. Flies have three Rocs: Roc1a is present in the fly Cullin1-based SCF complex and also binds Cullins2–4, while Roc1b binds Cullin3 and Roc2 binds Cullin5 [21]. This suggested that Roc1a should be required for regulating Arm. However, studies in *Drosophila* imaginal discs suggested that Roc1a does not negatively regulate Arm levels, though it does regulate levels of the Hedgehog effector Ci [6]. Subsequent work from the Duronio lab revealed that mutants lacking either Roc1b or Roc2 are adult viable, thus rendering it quite unlikely that they play a critical role in regulating Wnt signaling via Arm [21], [22]. Thus the identity of the RING finger protein in the SCF complex regulating Arm levels remained a mystery.

Using *Drosophila* cultured S2 cells, we found that Roc1a does play an important role in negatively regulating Arm levels, at least in that cell type. In contrast, neither Roc1b nor Roc2 RNAi increased Arm levels, and Arm levels were normal in *Drosophila* embryonic or larval tissues mutant for Roc1b or Roc2. Thus, it seems likely that Roc1a is the major Roc protein in the SCF complex regulating Arm levels. Why did previous work suggest otherwise? We believe this was due to the key role Roc1a plays in many different E3 ligases. Roc1a associates with Cullin1, Cullin2, Cullin3, and Cullin4 [21]. Consistent with it serving a critical role in many different cellular functions, Roc1a is essential for cell proliferation [6]. When clones of cells mutant for *Roc1a* are generated in imaginal discs, clones are only 1–3 cells in size, too small to assess Arm stability. This suggests that as Roc1 levels drop, cells rapidly stop proliferating, perhaps when residual Roc1a still remains from parental wild-type cells from which the clone of homozygous mutant cells was generated. To examine effects of Roc1a depletion on levels of Ci or Arm, Noureddine et al. generated clones of cells retaining a small amount of Roc1a function, by inducing production of Roc1a using a heat-shock promoter, allowing them to give clones of *Roc1a* mutant cells a pulse of Roc1a protein [6]. This allowed generation of larger clones, but left the caveat that cells in these clones begin with elevated Roc1a levels that decay over time. We hypothesize that the threshold for Roc1a function in Arm stability is lower than that for cell cycle progression or Ci stability. In this model, cells arrested before SCF function was compromised enough for Arm levels to rise. Of course, it remains possible that the role of Roc1a in Arm degradation is cell type specific, with S2 cells requiring it and imaginal disc cells not doing so.

Our RNAi screen also assessed other potential SCF complex proteins. Consistent with previous data [4], [5] and with the known composition of the canonical SCF complex, Cullin1 and SkpA scored positive in our screen, as did the F-box protein Slimb. However, we did not find a role for Cullin4, as was previously suggested [10], nor did the F-box protein Ebi, implicated in regulating ßcat levels [7], [8], score positive in this cell type. Of course, those proteins may have cell type specific roles in Arm/ßcat regulation, but they are less likely to have general roles in this process.

### Similar machinery regulates Arm levels in embryonic and larval tissues

We also addressed whether machinery regulating Arm levels differs in embryonic or larval tissues. In *Drosophila* embryos, inactivating any component of the destruction complex, including both APC family members, Axin, or the kinase GSK3 leads to highly elevated Arm levels [12]–[17]. However, data from larval tissues was puzzling, as clones of cells mutant for both APC proteins in the larval brain only accumulated modest levels of Arm [18]. This raised the possibility that different proteins regulate Arm in different tissues.

We thus analyzed, in parallel, clones of wing imaginal disc cells mutant for the destruction complex proteins APC1 plus APC2 or Axin, or for the SCF proteins Slimb or Cullin1. Most clones mutant for each of these genes accumulated modest levels of Arm. Some clones did appear to accumulate much higher levels of Arm. However, because cells in wing imaginal discs with activated Wnt signaling apically constrict and invaginate [27], confocal sections through discs with clones of mutant cells do not always pass through the same part of the cell in mutant cell clones and wild-type neighbors. Since Arm is a component of cell-cell adherens junctions, a section through the apical end of a cell will reveal much higher Arm levels, as it will pass through adherens junctions. When we controlled for this, similar modest increases in Arm levels were seen in all genotypes we analyzed.

However, these data do suggest that Arm levels rise more dramatically when the destruction complex is inactivated in embryos relative to imaginal discs. Our data also provide a possible explanation for this. *arm* is transcriptionally upregulated at the mid-blastula transition [30]. Our data suggest that stage 9 embryos, when Wnt signaling is maximally active in the embryonic epidermis [37], have 2–3 fold more *arm* mRNA than imaginal disc cells—this was apparent both by Northern analysis and from RNAseq data. However, Arm protein levels in the two tissues are similar or even opposite, suggesting the destruction complex simply destroys any excess Arm programmed by the higher mRNA levels in embryos. In fact, the destruction complex can handle levels of Arm protein higher than those normally seen in embryos, as overexpressing wild-type Arm using the GAL4-UAS system has no apparent consequences for Wnt signaling [38].

However, if translation rates are similar in embryonic and larval tissues, the elevated levels of *arm* mRNA in stage 9 embryos would mean that inactivating the destruction complex would lead to more rapid increases in Arm levels in embryos than in imaginal discs, as is observed. This might make sense, as Wingless signaling in the embryonic epidermis is highly dynamic, with multiple roles in the span of just a few hours [37] and rapid evolution of the pattern of ligand expression [39]. Having elevated levels of *arm* mRNA would facilitate more rapid increases in Arm protein levels in response to dynamic Wingless signaling. It is also curious that loss of destruction complex proteins in embryos leads to much higher accumulation of Arm than is seen in wild-type embryonic cells that receive Wnt signals. This may suggest that the levels of Wnt signaling experienced by embryonic cells do not fully inactivate the destruction complex—this is, of course, only speculative.

### An APC2 protein lacking the Arm repeats retains residual ability to limit Wnt signaling

The mechanisms by which APC proteins act in the destruction complex remain incompletely understood. One issue concerns the role of the N-terminal Arm repeats. Data from mammalian cells initially suggested that this region of APC might be dispensable, as fragments of the central region of APC lacking the Arm repeats rescued Arm destruction in cultured human colon cancer cells [40]. However, the endogenous copy of *APC* in these cells encodes a truncated APC protein retaining the Arm repeats; this might complement the other APC fragment in trans. In contrast, in *Drosophila* several point mutants in the Arm repeats reduce or eliminate APC2 function in Wnt regulation [32]. Here we tested the role of the Arm repeats in APC2 function directly, creating a mutant, APC2ΔArmrepeats, which cleanly deletes them.

Unlike full-length APC2 [36], APC2ΔArmrepeats cannot rescue ßcat destruction in SW480 cells, suggesting it cannot rescue function of the destruction complex. Consistent with this, APC2ΔArmrepeats had little ability to rescue Wnt signaling defects of *Drosophila* embryos lacking both APC1 and APC2. However, APC2ΔArmrepeats could provide substantial rescue of Wnt signaling defects in embryos lacking APC2 but retaining APC1. Further, in SW480 cells, APC2ΔArmrepeats could partially reduce Wnt-responsive transcription of a reporter gene. Together with our previous analysis of other *APC2* mutants [36], we thus favor the hypothesis that APC2ΔArmrepeats, because it retains multiple ßcat binding sites, can reduce Wnt signaling by binding to and sequestering ßcat, thereby reducing transcriptional activation of Wnt target genes. APC2Armrepeatsonly, in contrast, had little or no rescuing ability either in *APC2* single mutants or in SW480 cells, suggesting that it retains little or no function in Wnt regulation.

These data are also of interest because they cast further light on an interesting *APC2* mutant, *APC2^33^*, previously characterized by Takacs et al. (2008) [19]. *APC2^33^* was isolated as part of a screen for genetic modifiers of the phenotype of fly *APC1* mutants, in which Wnt signaling is inappropriately activated in the developing eye, leading to massive apoptosis. Surprisingly, heterozygosity for deletions removing *APC2* suppressed the apoptosis caused by loss of APC1. This suggested the paradoxical hypothesis that APC2 plays positive as well as negative roles in Wnt signaling. Takacs et al also generated two deletion alleles of *APC2* by mobilizing P element transposons inserted upstream [19]. One, *APC2^19-3^*, deleted almost the entire coding sequence, extending through the second 20 amino acid repeat (Fig. 6A), while the other, *APC2^33^*, deleted N-terminal coding sequence, extending most of the way through the sequences encoding the Arm repeats (Fig. 6A). Both alleles suppressed the eye phenotype of *APC1*. In contrast, the allele our lab generally uses as its null allele, *APC2^g10^*, which has a stop codon in the seventh Arm repeat (Fig. 6A) and which our immunoblotting suggests doesn't encode a stable protein [32], did not suppress *APC1's* eye phenotype.

To explain why some alleles suppress loss of APC1 and others do not, Takacs et al. hypothesized that the putative positive role of APC2 requires the N-terminal Arm repeats [19]. This hypothesis suggests that both *APC2^33^* and *APC2^g10^* encode stable truncated proteins, the former lacking the N-terminal Arm repeats and the latter lacking everything C-terminal to the Arm repeats (in neither case could this truncated protein be detected with the relevant antibody [19], [32], so their levels must be very low). Both their analysis in wing imaginal discs [19] and our data presented above support the hypothesis that *APC2^33^* retains some function in negative regulation of Wnt signaling. Further, by comparison with APC2ΔArmrepeats, our data provide a mechanistic hypothesis for how it does so. However, our data also point out that there is not a correlation between an allele's degree of defect in negative Wnt regulation and its function in suppressing loss of APC1. Both *APC2^19-3^* and *APC2^g10^* have strong defects in Wnt regulation, yet only one suppresses loss of APC1.

It would thus be worth re-visiting the mechanisms by which APC2 (or at least some APC2 mutant proteins) exert their positive role in Wnt signaling. The array of new alleles available from our site-directed mutagenesis [36], plus the alleles described here, would facilitate a detailed analysis of what domains are required for APC2's paradoxical positive role in Wnt signaling, and thus the mechanisms by which it acts in this process.

## Materials and Methods

### Fly Stocks and transgenic constructs

All experiments were done at 25°C. Mutations and Balancer chromosomes are described at FlyBase (flybase.bio.indiana.edu). Fly APC2 constructs and transgenic flies were generated as described in [36]. Briefly, sequences encoding full-length *Drosophila* APC2 (amino acids 1–1067), APC2Armrepeatsonly (aa 1–465), or APC2ΔArmrepeats (aa 466–1067) were PCR amplified and cloned into the Gateway entry vector pCR8/GW/TOPO (Invitrogen). Gateway recombination was then performed according to the manufacturer's instructions (Invitrogen) into appropriate destination vectors. For mammalian cell culture, this was a modified ECFP N1 vector (Clonetech) with an added GFP-Gateway cassette. To generate transgenic flies, *APC2* constructs were Gateway cloned into a modified pUAStattB vector (Basler lab, GenBank accession number EF362409) that added the endogenous *dAPC2* promoter [32] and an EGFP-Gateway-3× STOP cassette. Additional details of cloning are available upon request. Transgenic lines were generated by Best Gene Inc. (Chino Hills, CA) using PhiC31 integrase-mediated transgenesis at genomic position 28E7 (BDSC Stock# 9723).

### Fly crosses

Transgenes were crossed into *APC2^g10^* single mutant or *APC2^g10^ APC1^Q8^* double mutant backgrounds as previously in [36]. Progeny that expressed the transgene but were maternally/zygotically mutant for endogenous *APCs* were analyzed for embryonic lethality and cuticle rescue. Previously established criteria were used to score embryonic cuticle rescue [32]. Cuticle preparations were as in [41].

### Generating Mutant Clones

Clones were generated by FLP/FRT mediated mitotic recombination using the MARCM strategy [42], except for *Cullin1* clones, which were generated by a standard GFP-negative approach. Briefly, *FRT82B APC2^g10^ APC1^Q8^* females (or female flies with analogous mutations) were crossed to *y w hsflp1*, *UASmCD8::GFP; tubGAL4; FRT82B tubgal80/TM6b Tb* males. Clones were induced by a 3 hr heat shock at 37°C, 2 and 3 days after egg laying. After heat shock, larvae were returned to 25°C for two days. Female, non-Tubby, 3rd instar larva were dissected and analyzed for clones. For GFP negative clones, *y w hsflp12; FRT42D UbiGFP/CyO* females were crossed to *FRT42D Cul1^Ex^/CyO* males. Female GFP-positive larvae were collected and dissected.

### Immunofluorescence

#### *Drosophila* embryos and larval tissues

We used mouse monoclonal anti-Armadillo7A1 (Developmental Studies Hybridoma Bank). GFP-labeled proteins were detected by GFP-fluorescence. Embryos were collected for two hours at 25°C, and then let age 5 hours (to stage 9). For larval collections wandering 3rd instar larvae were dissected, and brains and wing discs loosened from the cuticle to allow easier antibody access. Embryos were fixed 20 minutes in 10% formaldehyde in phosphate-buffered saline (PBS). Larval tissues were fixed 20 min in 4% formaldehyde in PBS. All were blocked 30 min in 1% normal goat serum and 1% Triton X-100 in PBS (PBT). Antibodies were diluted in PBT as follows: α-Arm 1∶50, for larval brains and wing discs, 1∶100 for embryos, Alexa-labeled α-mouse secondary antibody (Molecular Probes) 1∶250. Primary antibodies were incubated at 4°C overnight, and secondary antibodies were incubated three hours at 25°C. Prior to mounting brains and wing discs were dissected completely from the cuticle. All samples were mounted in Aqua Poly/Mount (Polysciences). Fixed samples were imaged with a Pascal confocal microscope, using a Zeiss 40× NA 1.3 Plan- Neofluar oil immersion objective, and LSM software at 25°C. Adobe Photoshop CS2 was used to adjust input levels so the main range of signals spanned the entire output grayscale and to adjust brightness and contrast.

#### SW480 cells [40]

Cells were plated on sterile glass coverslips and transfected with various *APC2* constructs. 24 hours post-transfection, cells were fixed 5 min in 4% formaldehyde/1× phosphate-buffered saline (PBS), blocked with 1% normal goat serum (NGS)/0.1% Triton-100/1× PBS, and then antibody stained. The βcat antibody (cat# sc-7199; Santa Cruz Biotechnology, Santa Cruz, CA) was used at 1∶800.

### Immunoprecipitations and Immunoblotting

#### *Drosophila*

Embryos were collected for two hours and aged 5 hours (stage 9), or 21 hours (stage 17). Brains and wing discs were dissected from wandering 3rd instar larvae. All samples were boiled 5 min in 2× Laemmli buffer, run on 8% acrylamide gels and transferred to nitrocellulose membrane. Blots were incubated one hour with α-Arm (1∶75), along with α-tubulin (DM1A, 1∶7500, Sigma) or anti-Peanut (1∶50; DSHB) as loading controls. Washes were in Tris-Buffered Saline Tween-20 (TBST) at 4×15 min. For detection, blots were incubated one hour with horseradish peroxidase-conjugated rabbit α-mouse IgG secondary antibody (1∶20000, Zymed), and then the ECL-Plus kit (GE Healthcare Amersham) was used. For immunoprecipitations, dechorionated embryos were first lysed in NET buffer (50 mM Tris, pH 7.5, 400 mM NaCl, 5 mM EDTA, 1% NP40) containing protease inhibitors (Complete EDTA-free Protease Inhibitor tablets; Roche, Basel, Switzerland) and phosphatase inhibitors (1 mM NaF, 0.4 mM NaVO3). Antibodies were anti-GFP (JL-8; Clontech) at 1∶200, mHRP (1∶5000).

#### S2 cells

RNAi treated S2 cells were collected and pelleted by brief centrifugation. Cell pellets were resuspended in 1× PBS+0.1% TritonX-100 and a small sample removed to determine protein concentration via Bradford. 2× Laemmli buffer was then added, the samples boiled for 5 mins, and lysates analyzed by immunoblotting as described above.

### Cell culture, RNAi, Transfections

*Drosophila* S2 cell cultures were maintained as described [43]. Gene specific primers were used to generate dsRNA for target genes (500–1000 bp in length; specific primer sequences are available upon request). RNAi was performed in 6 well plates by treating near confluent cells with 10 µg dsRNA in 1 ml of fresh media every day for 7 days. On day 4, the cells were re-plated in a new well to maintain appropriate cell density. SW480 cells were cultured, transfected with Lipofectamine 2000 (Invitrogen) per manufacturer's protocol, and analyzed as previously described [36].

### High-Throughput Microscopy

S2 cells were seeded in concanavalin A-coated 24-well glass-bottom plates (Greiner) for 1 hour prior to fixation, fixed with 10% formaldehyde, stained with anti-Arm monoclonal antibody and Hoechst (Invitrogen), and scanned with an Array Scan VTI (Cellomics) equipped with a 20× 0.5 NA or 40× 0.95 NA objective and an ORCA-ER cooled CCD camera. Images of ∼2,000–10,000 cells per well were acquired and analyzed using vHCS View (Cellomics). Integrated fluorescence intensity measurements were determined from unsaturated images.

### Northern Blotting

RNA was isolated with TRIzol (Sigma-Aldrich) from embryos (stage 9 or stage 17) and brains and wing discs from 3rd instar larvae according to manufacturer's directions. 3 µg of each sample were fractionated on a 1.5% agarose-formaldehyde gel and then this was transferred to a nylon membrane. Prehybridization, hybridization, and posthybridization washes were done as described in [44]. Hybridization was at 60°C. Probes for each transcript were made radiolabeling using either T7 (*rp49*, New England Biolabs) or T3 (*arm*, Promega) polymerases as in [45]. The membrane was simultaneously probed with riboprobes for *arm* and *rp49* as an internal control.
